# Atypical Presentation Revealing Sorsby Macular Dystrophy: A Case Report

**DOI:** 10.7759/cureus.57976

**Published:** 2024-04-10

**Authors:** Taha Boutaj, Hamza Lazaar, Abdellah Amazouzi, Samira Tachfouti, Lalla Ouafa Cherkaoui

**Affiliations:** 1 Ophthalmology, Hospital des Specialités de Rabat, Rabat, MAR; 2 Ophthalmology, Mohammed V University, Hospital des Specialités de Rabat, Rabat, MAR

**Keywords:** drusen, anti-vegf, dystrophy, macula, sorsby

## Abstract

Sorsby macular dystrophy is an autosomal dominant disorder secondary to heterozygous mutations in the TIMP3 gene in 22q12. It begins with fine, pale, drusen-like deposits or confluent, faint yellow material or sheets beneath the retinal pigment epithelium, but it eventually progresses to either geographic atrophy with pigmentary clumps or scars due to the choroidal neovascular membrane around the fourth decade of life. We describe a patient who presented with a progressive loss of unilateral visual acuity, wrongly suggesting an infectious or inflammatory disease.

## Introduction

Sorsby macular dystrophy is an autosomal dominant disorder with complete penetrance secondary to heterozygous mutations in the TIMP3 gene in 22q12 [1]. The mechanisms surrounding Sorsby macular dystrophy remain incompletely understood. While Sorsby and other researchers suggested an inflammatory basis for the disease [2], Duke-Elder introduced the term "Sorsby’s pseudo-inflammatory fundus dystrophy" in light of his skepticism regarding the inflammatory pathway, thus providing a nuanced scientific perspective on the condition [3]. It is characterized by the development of choroidal, retrofoveal neovascularization, often bilateral, usually around the age of 40 [1]. It progresses to chorioretinal atrophy [4]. Its sudden onset is often mistaken for an acute inflammatory disease.

We report the case of a 43-year-old woman who presented with a progressive loss of unilateral visual acuity, wrongly suggesting infectious or inflammatory disease.

## Case presentation

**Figure 1:**
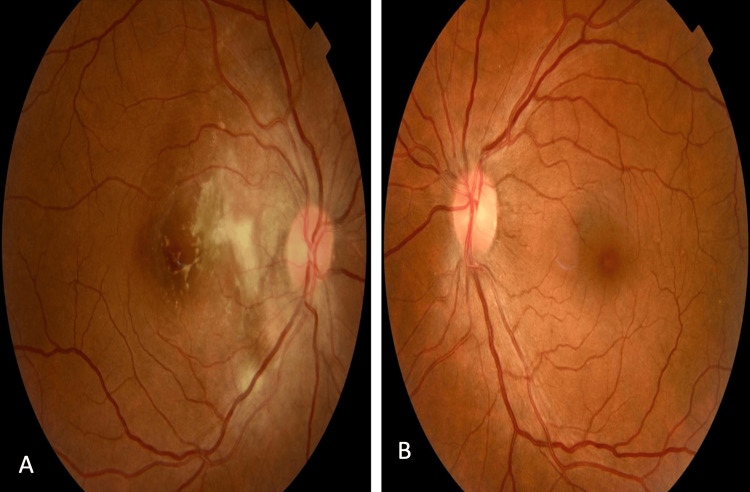
Funduscopy of both eyes showing macular dystrophy in the right eye (A) and drusens in the left eye (B)

We report a case of a 43-year-old woman with a strong family history of ‘poor vision’, who consulted for decreased visual acuity in her right eye over the last 6 months, with no other associated signs. On the ophthalmologic examination, the best corrected visual acuity was 1/10 in the right eye and 9/10 in the left one. The pupillary light reflex was present in both eyes. Intraocular pressure was normal. Slit-lamp biomicroscopic examination showed a clear cornea, a calm anterior segment with the filamentous vitreous, and a clear lens without cataract. Funduscopy of the right eye revealed a grayish lesion with irregular edges, taking up the papilla and invading the interpapillary-macular space, with a fuzzy white macular focus in this space. The left fundus showed yellowish deposits around the macula corresponding to drusens (Figure 1).

**Figure 2:**
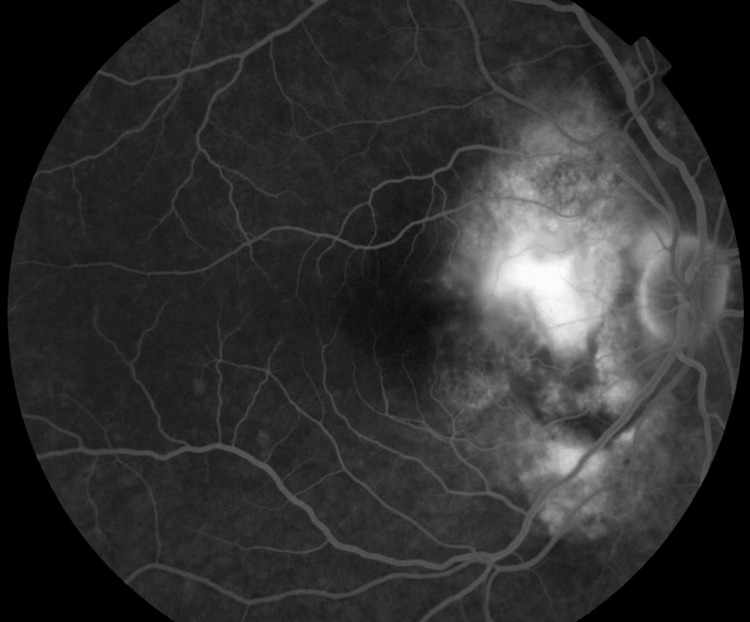
Fluorescein angiography of the right eye revealed a chorioretinal focus

Fluorescein angiography shows a hyperfluorescent area at the arteriovenous phase with increased fluorescence in the late phase (Figure 2).

**Figure 3:**
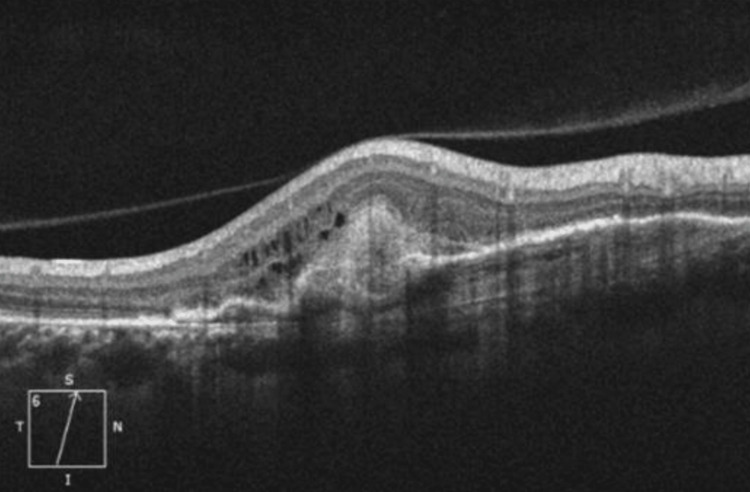
Macular OCT of the right eye revealed the presence of fibrosis at the focal point with macular edema and choroidal neovascularization OCT: optical coherence tomography

Macular optical coherence tomography (OCT) objectified the presence of fibrosis at the focal point, along with macular edema and choroidal neovascularization. Posterior vitreous detachment (PVD) was also present (Figure 3).

The color blind test, orbital MRI, and thoraco-abdominal CT scan were normal. Biological tests looking for infectious, inflammatory, or immune pathologies were also normal. A genetic test was conducted showing a mutation in exon 5 of the TIMP3 gene.

The patient received eight injections of anti-vascular endothelial growth factor (Bevacizumab) in the right eye. No treatment was initiated for the left eye.

The final visual acuity in the right eye was 4/10 after a year. The patient continues to be monitored in our facility in case of a recurrence of neovascularization.

## Discussion

First described in 1949 in five families and with a prevalence of 1/220 000 [5], Sorsby dystrophy is a retinal dystrophy that can manifest abruptly at the age of 40 to 50 as a unilateral hemorrhagic and exudative maculopathy.

It usually affects the second eye and, without treatment, will progress to a disciform scar [6]. The sudden installation is often confused with inflammatory and infectious diseases. It is secondary to heterozygous mutations in the TIMP3 gene on 22q12. Mutations usually affect exon 5 [6]. This gene encodes a tissue inhibitor of metalloproteinase, which is involved in the remodeling of the extracellular matrix [1]. This anomaly would lead to an accumulation of abnormal material under the pigment epithelium and thickening of the Bruch membrane, thus reducing its permeability to vitamin A, a precursor of the active chromophore (11-cis RAL) [6].

Recent studies have demonstrated the inhibitory effect of TIMP3 on angiogenesis mediated by vascular endothelial growth factor (VEGF) [7]. TIMP3 prevents the binding of this growth factor to the receptor, VEGFR2. This property, independent of metalloproteinase activity, would explain the observed submacular choroidal neovascularization.

The main sign motivating the consultation is the progressive loss of visual acuity. This loss is often bilateral and asymmetric, revealing itself around the age of 40. In our case, it occurred only in the right eye, but the drusen present in the left eye suggests the beginning of dystrophy. Besides the loss of visual acuity, other ophthalmologic signs can be seen as a disturbance of night vision or an impairment of color vision. In 34 asymptomatic patients from three families at a 50% risk of developing Sorsby fundus dystrophy, color contrast sensitivity was measured. In 16, the thresholds - mainly on the Tritan axis - were raised above the normal values. It is concluded that testing color vision is useful in detecting the abnormal genotype [8].

There is currently no proven treatment for Sorsby fundus dystrophy. Argon laser therapy and photodynamic therapy (PDT) are not effective [9]. Anti-VEGF injections have been shown to be effective in controlling the growth of new blood vessels [10].

Sorsby fundus dystrophy needs to be diagnosed early and confirmed by genetic testing. This means we can give people a better idea of how their sight loss is likely to develop and look out for the symptoms of new blood vessels growing in the retina.

## Conclusions

Sorsby macular dystrophy is a rare, inherited condition that starts suddenly around the age of 40. It is secondary to a heterozygous mutation in the TIMP3 gene. It must be diagnosed early and confirmed by genetic testing, as a genetic study can highlight this anomaly. Anti-angiogenic injections have been shown to be effective.
